# Machine learning-based prediction of post-induction hypotension: identifying risk factors and enhancing anesthesia management

**DOI:** 10.1186/s12911-025-02930-y

**Published:** 2025-02-22

**Authors:** Ming Chen, Dingyu Zhang

**Affiliations:** 1 Department of Anesthesiology, Union Hospital, Tongji Medical College, Huazhong University of Science and Technology, Wuhan, 430022 China; 2 Department of Anesthesiology, Institute of Anesthesia and Critical Care Medicine, Union Hospital, Tongji Medical College, Huazhong University of Science and Technology, No. 1277, Jiefang Avenue, Wuhan, 430022 China; 3 Wuhan Jinyintan Hospital, Wuhan, 430023 China

**Keywords:** Post-induction hypotension, Machine learning, Logistic regression, Risk factors, Personalized medicine

## Abstract

**Background:**

Post-induction hypotension (PIH) increases surgical complications including myocardial injury, acute kidney injury, delirium, stroke, prolonged hospitalization, and endangerment of the patient's life. Machine learning is an effective tool to analyze large amounts of data and identify perioperative complication factors. This study aims to identify risk factors for PIH and develop predictive models to support anesthesia management.

**Methods:**

A dataset of 5406 patients was analyzed using machine learning methods. Logistic regression, random forest, XGBoost, and neural network models were compared. Model performance was evaluated using the area under the receiver operating characteristic curve (AUROC), calibration curves, and decision curve analysis (DCA).

**Results:**

The logistic regression model achieved an AUROC of 0.74 (95% CI: 0.71–0.77), outperforming the random forest (AUROC: 0.71), XGBoost (AUROC: 0.72), and neural network (AUROC: 0.72) models. In terms of calibration, logistic regression demonstrated superior performance, as reflected by Brier Scores and calibration curves, followed by XGBoost, random forest, and neural network. Decision curve analysis indicated that the logistic regression model provided the greatest clinical utility among all models. Baseline blood pressure, age, sex, type of surgery, platelet count, and certain anesthesia-inducing drugs were identified as important features.

**Conclusions:**

This study provides a valuable tool for personalized preoperative risk assessment and customized anesthesia management, allowing for early intervention and improved patient outcomes. Integration of machine learning models into electronic medical record systems can facilitate real-time risk assessment and prediction.

**Supplementary Information:**

The online version contains supplementary material available at 10.1186/s12911-025-02930-y.

## Introduction

Post-induction hypotension (PIH) is a common yet perilous adverse effect, posing an increased risk of surgical complications, including myocardial injury, acute kidney injury, delirium, stroke, prolonged hospital stay, and jeopardizing the patient's life [1–4]. After induction of anesthesia, anesthesiologists are occupied with tasks such as tracheal intubation, adjusting anesthetic drug dosage, fine-tuning ventilator settings, and documenting medical records, which could potentially lead to the oversight of PIH. Therefore, it would be beneficial to accurately predict the risk of PIH and its associated risk factors in advance.

Machine learning, being potent predictive tool, has exhibited a broad spectrum of applications in the medical domain. Through comprehensive analysis of extensive preoperative and intraoperative data, machine learning models have the capability to identify significant factors linked to the occurrence of perioperative complications. The seamless integration of machine learning models into electronic medical record systems holds the potential to facilitate real-time risk assessment and prediction, thereby enhancing patient care and outcomes.

However, current models for PIH prediction often rely on complex machine learning algorithms and rigorous data collection methods, making them susceptible to overfitting issues, especially when the dataset has limited patient samples. For instance, incorporating invasive arterial pressure data into the analysis may improve prediction accuracy, but such data are only available for high-risk patients in specific procedures and cannot be generalized to the broader patient population. Non-invasive methods like the pleth variability index (PVI), derived from pulse oximetry, provide insights into fluid responsiveness but require specific pulse oximeter devices [5]. Similarly, heart rate variability (HRV), obtained via electrocardiograms (ECG), adds analytical complexity, making it less suitable for widespread use [6]. Ultrasonography, used to assess subclavian or axillary veins, offers another non-invasive approach but depends on the availability of ultrasound equipment [7]. In contrast, this study leverages routinely collected data from electronic medical records (EMRs) to predict PIH, eliminating the need for specialized diagnostic tools. We utilized the VitalDB open dataset, which encompasses routine clinical data, and applied multiple machine learning methods for modeling. We evaluated the models based on discrimination, calibration, and clinical applicability, aiming to enhance their practical implementation and interpretability in clinical settings.

This study aims to identify risk factors for PIH and enhance patient outcomes through the utilization of machine learning models. In cases where patients are at a higher risk, anesthesiologists can modify the anesthetic protocol, implement proactive fluid management strategies, and adjust medication dosages to mitigate the occurrence of PIH. By leveraging these models, anesthesia teams are provided with a practical tool for personalized preoperative risk assessment and tailored anesthesia management.

## Methods

### Data source

In this study, we obtained the data from VitalDB, a publicly available repository that gathered biosignal and clinical information from 6388 surgical patients during their surgeries [8]. The data covers the period from January 2005 to January 2014 and includes patients undergoing non-cardiac surgeries, such as general, thoracic, urologic, and gynecologic procedures. The dataset contains biological information, including blood pressure, heart rate, and ventilator parameters, as well as clinical information, such as patient age, gender, BMI, type of surgery, and preoperative laboratory test results. This study has been reported in line with the STROCCS criteria [9].

### Data pre-processing

To process the preoperative and intraoperative data, we applied systematic feature engineering techniques. Ordered categorical variables were transformed using label encoding to maintain their inherent order. Multicategory variables were converted to binary representations via one-hot encoding. Continuous variables were standardized using z-scores to ensure uniform scaling across features. Variance filtering was conducted to exclude features with low variability (variance < 0.01), as these contribute minimally to model prediction. Correlation coefficient analysis was performed to remove highly correlated features (|r|> 0.8), thereby reducing multicollinearity and enhancing model interpretability.

PIH was defined as a mean arterial pressure (MAP) less than 55 mm Hg between the induction of anesthesia and the start of surgery. This threshold was based on previous studies that have identified a correlation between MAP less than 55 mm Hg and postoperative adverse events [4, 10, 11]. Baseline blood pressure was determined using the first noninvasive blood pressure measurement recorded upon operating room admission, prior to anesthesia induction. This ensures accurate baseline values without influence from anesthetic agents, reducing the risk of data leakage. To enhance data reliability, measurements outside the physiological range (MAP < 20 mmHg or > 160 mmHg) were excluded.

### Missing value processing

In our study, we employed median imputation to handle missing values in the dataset. This approach preserves the information content of the features without significantly reducing the sample variance, unlike mean imputation. Median imputation is not influenced by the dominant group within the features and better maintains the expression of the features, particularly when the number of missing values is relatively small. We conducted a sensitivity analysis to compare the performance of the model using median imputation with that of the model where missing values were directly removed.

### Machine learning models

In our study, we employed several machine learning models for prediction, including logistic regression, random forest, XGBoost, and neural network models. These models are widely used in classification problems, each with its own unique advantages and disadvantages. To ensure the accuracy and stability of the models, we divided the dataset into training and validation sets in a 7:3 ratio and utilized a five-fold cross-validation method. During the model training process, we performed grid search to fine-tune the model parameters, aiming to enhance the prediction accuracy and generalization capability of the models.

### Model performance and evaluation

To evaluate the performance of the models, we utilized several evaluation metrics. The discrimination of the models was assessed using the area under the subject operating characteristic curve (AUROC). AUROC was chosen as the primary metric for its ability to evaluate model discrimination across all thresholds without being affected by class imbalance, a key advantage in clinical datasets. Other metrics, including accuracy, precision, recall, and F1-score, were calculated to provide a more nuanced performance assessment. The thresholds for all models were selected using the maximum Youden’s index to ensure a consistent approach for optimizing sensitivity and specificity. The 95% confidence intervals for performance metrics were calculated using the bootstrap method.

Calibration performance was evaluated to assess the agreement between predicted probabilities and observed outcomes. A calibration curve was plotted by comparing predicted probabilities to observed event rates in deciles of predicted risk. A well-calibrated model should align closely with the diagonal reference line. Models were assessed using fivefold cross-validation, and comparisons were made based on visual inspection of calibration curves and Brier Scores.

To determine the clinical utility of the models, decision curve analysis (DCA) was performed. Net benefit is calculated as follows:

$$Net Benefit=\frac{TP}{N}-\frac{FP}{N}\times \frac{p}{1-p}$$

where TP and FP represent true positives and false positives, N is the total sample size, and p is the threshold probability.

DCA evaluates the net benefit across different threshold probabilities, reflecting the trade-offs between true positives and false positives in a clinical context. This metric helps in understanding the practical implications of implementing the predictive models in real-world clinical decision-making.

By considering these evaluation metrics, we aimed to assess the predictive ability, stability, and applicability of the models. Ultimately, these assessments allowed us to identify the best-performing machine learning model, which was selected as the final predictive model.

## Results

### Dataset characteristics

**Fig. 1:**
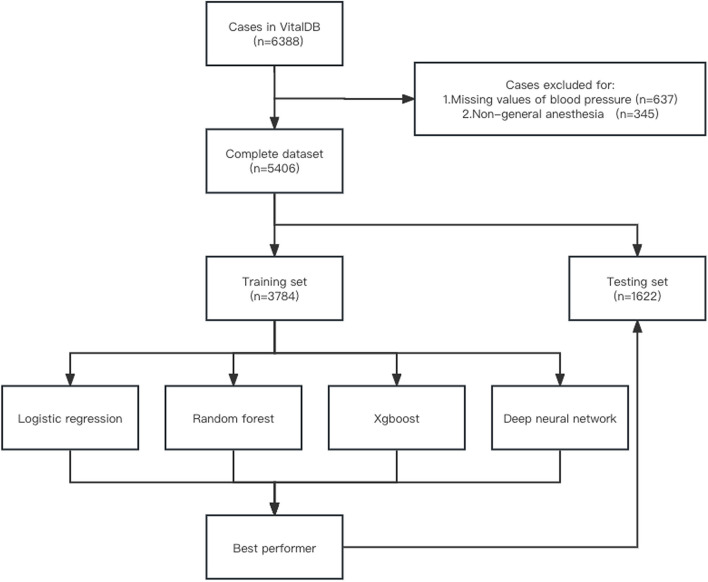
Patient recruitment flowchart

A total of 5,406 patients were included in the study, of which 921 patients, accounting for 17% of the total, developed post-induction hypotension (Fig. 1).

We extracted 36 features from all clinical variables. Among these features, 13 had missing data. The percentage of missing data for each variable was less than 10%. Complete data without any missing values for all features were available for 88.6% of patients in the dataset.

The descriptive statistics of the clinical characteristics of patients with and without PIH in the dataset are presented in Table 1. Significant differences were found in most clinical characteristics between patients who developed PIH and those who did not. Patients who experienced PIH tended to be of advanced age, female, have a low BMI, and exhibit low preoperative hemoglobin and albumin levels.

**Table 1:** Baseline characteristics of patients with and without post-induction hypotension

Variables	PIH (*N* = 921)	non-PIH (*N* = 4485)	*P*
Patient Characteristics			
Age, mean (SD)	58.4 (15.5)	57.1 (14.4)	0.016
Sex			< 0.001
Female	567 (61.6)	2157 (48.1)	
Male	354 (38.4)	2328 (51.9)	
Heigh, cm	159.9 (10.4)	162.6 (9.1)	< 0.001
Weight, kg	58.5 (11.0)	62.2 (11.7)	< 0.001
BMI	22.8 (3.5)	23.5 (3.6)	< 0.001
ASA PS			0.07
I	291 (31.6)	1284 (28.6)	
II	507 (55.0)	2647 (59.0)	
III	96 (10.4)	449 (10.0)	
IV ~ VI	10 (1.1)	23 (5.1)	
Department			< 0.001
General surgery	796	3487	
Gynecology	25	154	
Thoracic surgery	93	744	
Urology	7	100	
EMOP			0.845
Yes	101	482	
No	820	4003	
HTN			0.013
Yes	250	1403	
No	671	3082	
DM			0.412
Yes	88	469	
No	833	4016	
Laboratory Tests			
Hemoglobin	12.6 (1.8)	12.9 (1.9)	< 0.001
Platelet	240.0 (82.4)	244.6 (83.4)	0.134
PT	99.7 (15.7)	101.0 (14.3)	0.024
APTT	32.7 (4.9)	32.7 (7.4)	0.803
Na	139.9 (3.0)	140.2 (2.8)	0.004
K	4.2 (0.4)	4.2 (0.4)	0.511
Glucose	115.3 (43.4)	115.3 (41.8)	0.998
Albumin	4.0 (0.5)	4.1 (0.5)	< 0.001
AST	28.5 (52.6)	29.9 (139.8)	0.771
ALT	26.3 (56.7)	27.9 (85.4)	0.601
BUN	15.9 (10.0)	15.6 (9.4)	0.317
Creatinine	0.9 (1.0)	1.0 (1.4)	0.035
Baseline Blood Pressure			
SBP	136.5 (27.0)	147.7 (25.2)	< 0.001
MBP	95.2 (17.0)	104.9 (16.7)	< 0.001
DBP	71.9 (11.9)	79.8 (11.9)	< 0.001
Use of anesthetic drugs			
Propofol	328 (35.6)	1794 (40.0)	0.013
Fentanyl	146 (15.9)	937 (20.9)	0.001
Rocuronium	912 (99.0)	4402 (98.1)	0.062

*Abbreviations*: *PIH* Post-Induction Hypotension, *BMI* Body mass index, *ASA PS* American Society of Anesthesiologists Physical Status, *EMOP* Emergency operation, *HTN* Hypertension, *DM* Diabetes, *PT* Prothrombin time, *APTT* Activated partial thromboplastin time, *ALT* Aminotransferase, *AST* glutamine aminotransferase, *BUN* Blood urea nitrogen, *SBP* Systolic blood pressure, *MBP* Mean arterial pressure, *DBP* Diastolic blood pressure

### Model performance

**Fig. 2:**
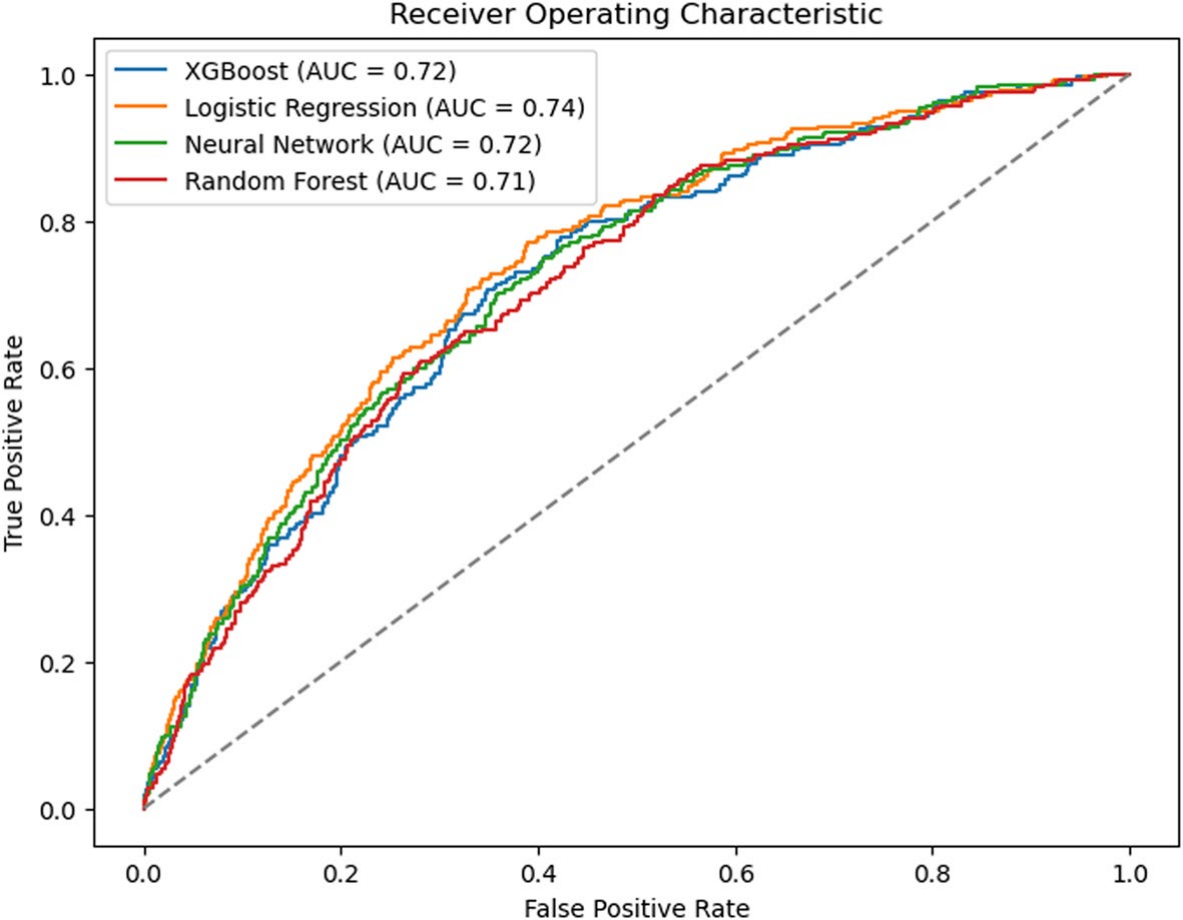
Receiver operator curves for the four machine learning models. Abbreviations: AUC, area under the curve

The AUROC was 0.74 (95% CI, 0.71—0.77) for the logistic regression model, 0.71 (95% CI, 0.68—0.74) for the random forest model, 0.72 (95% CI, 0.69—0.75) for the XGBoost model, and 0.72 (95% CI, 0.68—0.75) for the neural network model (Fig. 2). Differences between the four models were not statistically significant. Secondary metrics such as accuracy, precision, recall, and F1-score varied across models, with logistic regression maintaining a balanced trade-off (Table 2). Further sensitivity analysis of the missing value treatment method did not reveal any significant differences in performance improvement among the four models.

**Table 2:** Prediction performance of different machine learning models

	AUROC	Accuracy	Precision	Recall	F1-score
Logistic Regression	0.74 (0.71—0.77)	0.67 (0.64, 0.69)	0.31 (0.28, 0.35)	0.70 (0.64, 0.74)	0.43 (0.39, 0.47)
Random Forest	0.71 (0.68—0.74)	0.72 (0.70, 0.74)	0.34 (0.30, 0.38)	0.60 (0.55, 0.66)	0.44 (0.40, 0.48)
XGBoost	0.72 (0.69—0.75)	0.82 (0.80, 0.84)	0.65 (0.37, 0.91)	0.03 (0.01, 0.05)	0.06 (0.03, 0.10)
Neural Network	0.72 (0.68—0.75)	0.82 (0.81, 0.84)	0.65 (0.47, 0.83)	0.06 (0.04, 0.09)	0.11 (0.07, 0.16)

**Fig. 3:**
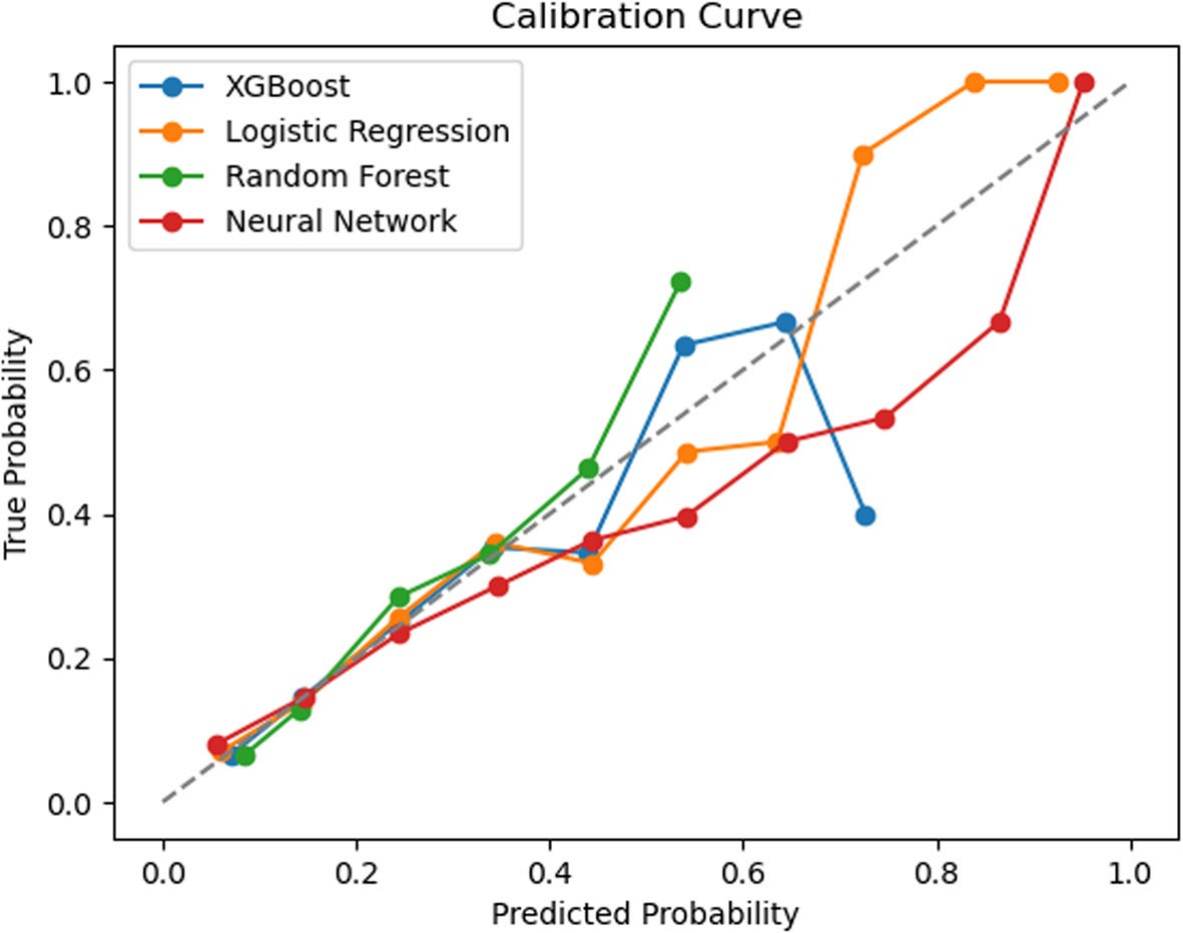
Calibration curves for for the four machine learning models

The calibration curves demonstrated that both the logistic regression and random forest models exhibited good calibration performance, indicating that the predicted probabilities aligned well with the observed probabilities. Brier Scores were as follows: logistic regression (0.1291), XGBoost (0.1305), random forest (0.1312), and neural network (0.1328). Based on Brier Scores and calibration curves, logistic regression displayed the best calibration, followed by XGBoost, random forest, and neural network (Fig. 3).

**Fig. 4:**
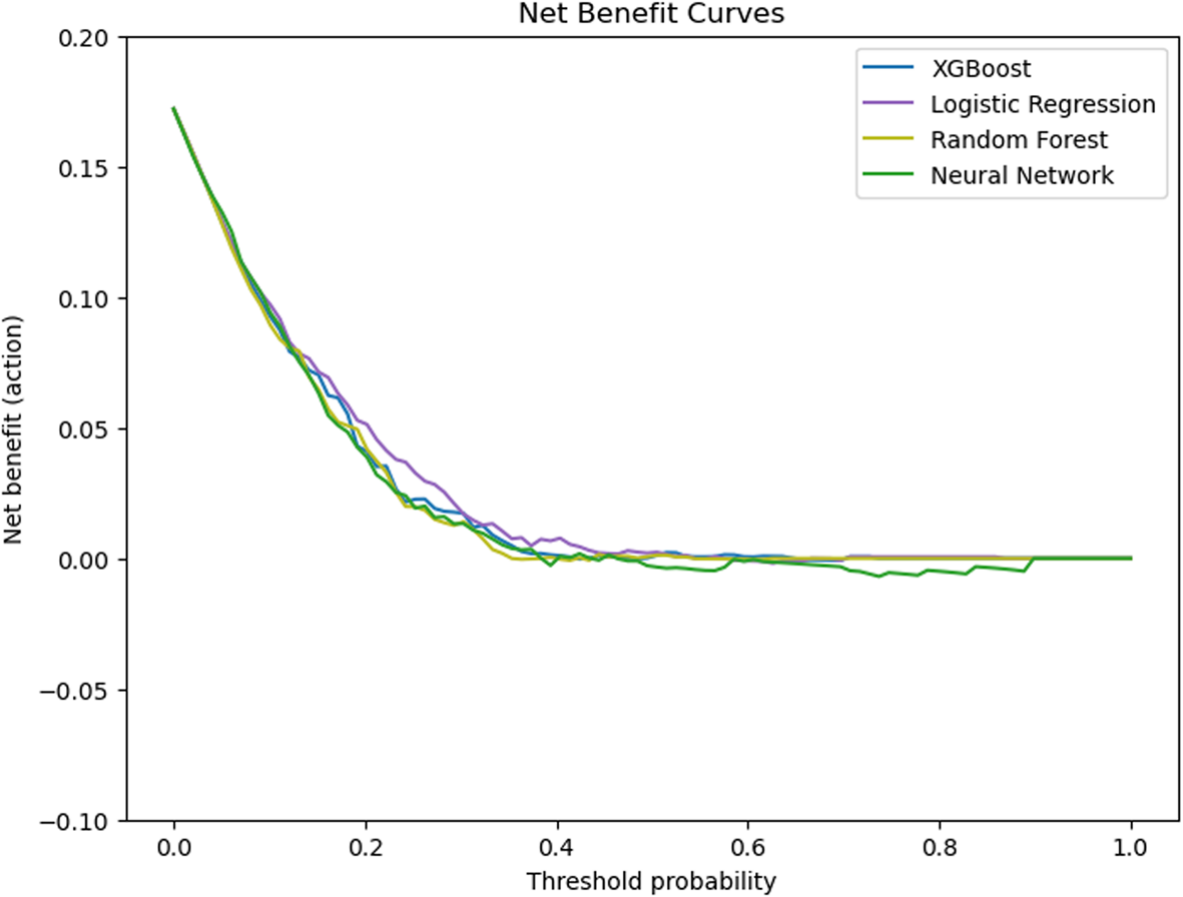
Decision curve analysis for the four machine learning models

Regarding the clinical benefit, the DCA curves indicated that the logistic regression model provided the highest clinical benefit compared to the other models (Fig. 4). This suggests that in the clinical prediction of PIH, logistic regression may offer a more favorable trade-off between reducing unnecessary interventions and avoiding missed diagnoses.

Considering the overall performance and interpretability, we selected the logistic regression model as the final prediction model for model interpretation.

### Model interpretation

**Fig. 5:**
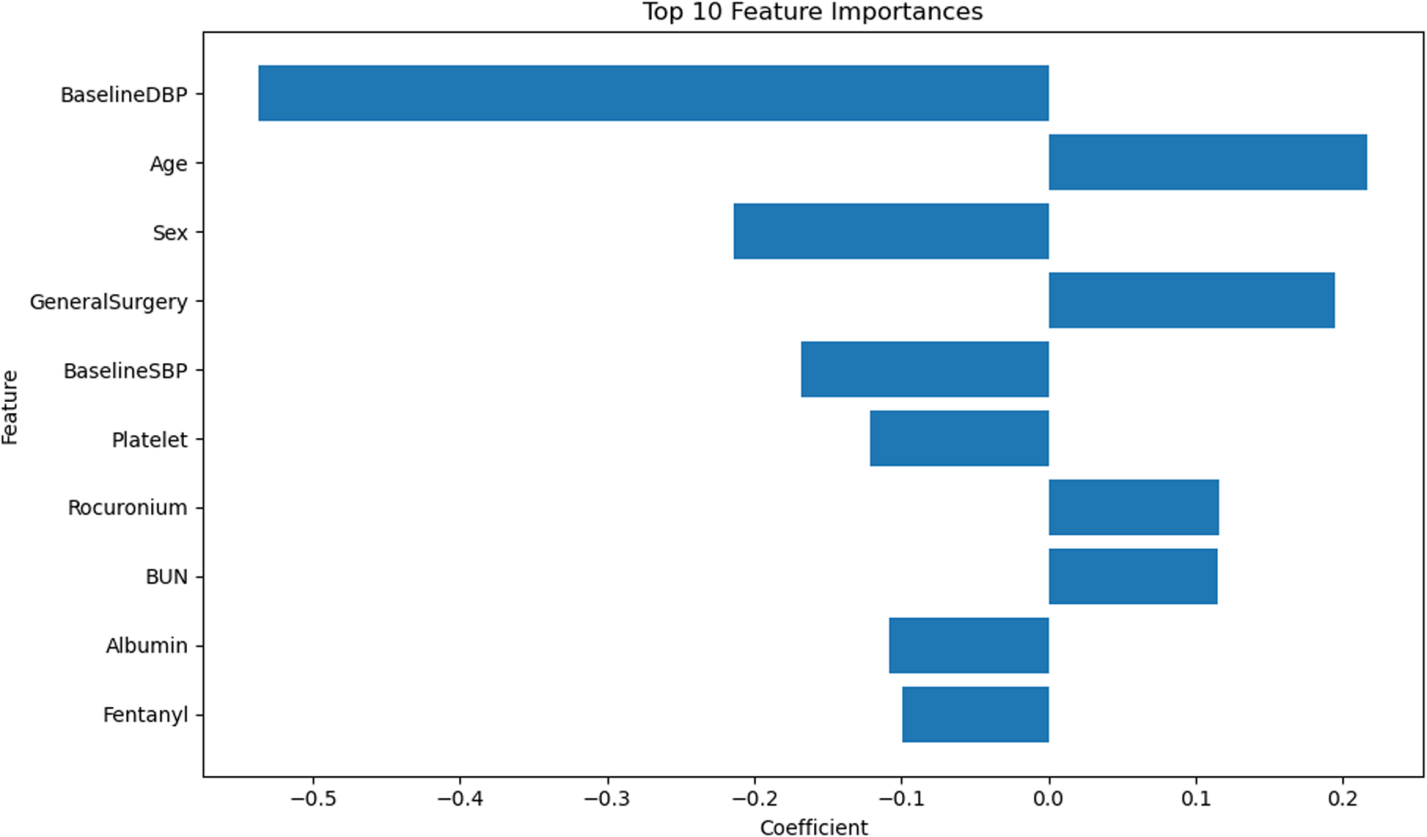
Coefficient value of the top features for the logistic regression model

The importance of features in the logistic regression model is determined by the absolute value of the feature coefficient which is intuitively interpretable. A larger coefficient value indicates a greater contribution of the feature to the model's prediction. By examining the magnitude of the coefficients, we identified the 10 most important features in the model (Fig. 5). These significant features, in descending order of importance, were baseline diastolic blood pressure, age, sex, type of surgery, baseline systolic blood pressure, platelet count, rocuronium bromide use, urea nitrogen, albumin, and fentanyl use.

## Discussion

This study developed a machine learning model to predict post-induction hypotension (PIH) using data from 5406 patients. Among the models tested, the logistic regression model demonstrated the best performance, achieving an AUROC of 0.74 (95% CI, 0.71 to 0.77). In previous studies, traditional logistic regression algorithms did not perform better than other machine learning models [12–15]. Models like XGBoost and neural network often outperform logistic regression in capturing non-linear relationships and interactions among variables [12]. However, the increased computational demands and the need for careful hyperparameter tuning may hinder its practical implementation in real-time clinical settings. Similarly, while random forest offers high predictive performance with minimal parameter tuning, it has been observed to be prone to overfitting prone to overfitting in smaller datasets or sparse data [15]. In this study, while logistic regression demonstrated comparable AUROC values to other models, its strengths in calibration, clinical utility and clinical applicability highlight its comprehensive performance. This result may be attributed to the class imbalance in the dataset, where more complex models are prone to overfitting the majority class, reducing their sensitivity to positive samples. Logistic regression, with its simpler structure, is considered the most sensitive classifier for imbalanced defect datasets [16], leading to more robust performance across metrics.

In decision curve analysis, the choice of threshold reflects the clinical preference for minimizing false positives versus false negatives. Given the nature of PIH, where overtreatment could pose unnecessary risks to patients and missed cases might delay timely interventions, selecting thresholds that favor higher precision may align better with clinical priorities. Future studies could explore different threshold ranges to evaluate whether alternative trade-offs might yield improved predictive utility under varying clinical contexts.

The results of the model interpretation provide insights into the significant features contributing to the prediction of PIH. One of the most important features is the baseline blood pressure, which aligns with previous research findings [13, 15, 17–20]. Higher baseline blood pressure tends to indicate a lower risk of hypotension during surgery, while lower baseline blood pressure may increase the risk of developing hypotension. However, it has also been suggested that high baseline blood pressure is a risk factor for PIH [21, 22]. The definition of outcome in these two studies was based on the percentage decrease in blood pressure relative to baseline, which may account for the diametrically opposed findings. Currently, there is no accepted definition of PIH, with studies employing thresholds of MAP < 55 mmHg, MAP < 60 mmHg, or MAP < 65 mmHg [23–25]. In this study, we chose MAP < 55 mmHg as a stricter threshold to focus on cases with more severe hemodynamic changes, which are more likely to have significant clinical implications. While this approach highlights severe PIH cases, we acknowledge that different thresholds might influence the reported incidence and clinical interpretation of PIH. Additionally, using baseline MBP alone to predict PIH showed an AUROC of 0.65, with poorer performance on calibration and DCA curves compared to comprehensive model (Supplementary materials).

Basic patient characteristics such as age and gender are found to potentially influence the occurrence of PIH. It is generally accepted that older patients are prone to PIH [6, 15, 17–19, 22], but there is controversy about the effect of gender [6, 12, 26]. Furthermore, laboratory indicators including platelet count, urea nitrogen, and albumin are identified as significant features. These indicators can reflect the patient's hematologic status and renal function. While direct evidence linking these indicators and hypotension during anesthesia is limited, abnormal values in these indicators may indicate disturbances in metabolic status and fluid balance, which could contribute to the occurrence of PIH. The use of anesthesia-inducing drugs affects patients' blood pressure, and incorporating this information into the model may help predict the risk of PIH. Although the use of rocuronium has no additional clinically relevant effects on cardiovascular dynamics, a transient decrease in blood pressure may be observed during the infusion [27, 28]. In contrast, fentanyl was negatively associated, likely because of its hemodynamic stability compared to other agents [29].

There are currently reported methods for predicting PIH using specialized equipment. For example, the pleth variability index, which is used to automatically estimate respiratory variability, has a sensitivity of 0.79 and a specificity of 0.71 for predicting PIH [5]. Heart rate variability analysis has an AUROC of 0.70 [7]. In addition, a model trained on vital signs recorded 4 to 1 min prior to intubation achieves an accuracy of up to 0.72 [20]. The model constructed in this study uses only data routinely collected from electronic medical records, eliminating the need for specialized diagnostic equipment or professional technical personnel. Although this approach may compromise accuracy, it strikes a balance between practicality and precision, providing a widely applicable and scalable tool for clinical risk stratification.

There are some limitations to the study. The use of a dataset from a single institutional database may introduce biases and limit the generalizability of the findings to other populations or healthcare settings. Second, the reliance on noninvasive blood pressure measurements rather than invasive measurements could introduce measurement errors and potential inaccuracies in capturing blood pressure dynamics. Another limitation is the lack of detailed information on vasoactive drug use. The dataset's limited information on the administration and dosing of vasoactive drugs may have affected the classification of outcome events and the predictive accuracy of the models. Additionally, the study mainly focused on preoperative features readily available in electronic medical records. While these features are easily accessible, not including additional intraoperative features, such as monitoring waveform data, intubation-related data, and ventilator parameters, may have limited the prediction of late PIH. To enhance the robustness and applicability of the predictive model, future studies could consider using multi-center datasets and incorporating more intraoperative features.

## Conclusions

This study provides a feasible machine learning model for predicting PIH and an insight into risk factors for PIH. The developed model can serve as a basis for future research and clinical practice, fostering advancements in personalized medicine and enhancing patient safety.

## Supplementary Information

Supplementary Material 1.

## Data Availability

The datasets generated and/or analysed during the current study are available in the VitalDB open dataset repository, https://vitaldb.net/dataset/.

## Abbreviations

PIH: Post-induction hypotension
MAP: Mean arterial pressure
AUROC: Area under the subject operating characteristic curve
DCA: Decision curve analysis
SHAP: Shapley additive explanation
