# Supplementary material for: Machine learning-based prediction of post-induction hypotension: identifying risk factors and enhancing anesthesia management
Source: BMC Med Inform Decis Mak. 2025 Feb 22;25:96. doi: 10.1186/s12911-025-02930-y (PMC11846375; doi:10.1186/s12911-025-02930-y)
Supplement: Supplementary file 1 — Supplementary Material 1. [file 12911_2025_2930_MOESM1_ESM.docx]

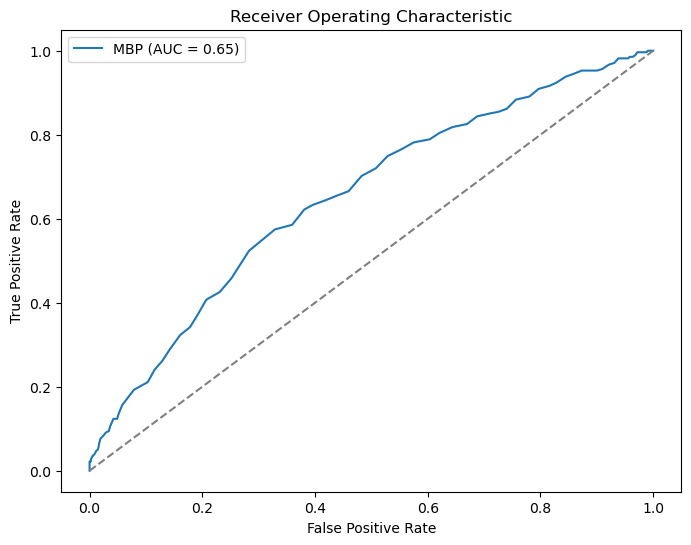


Fig. 1. Receiver operator curves for the baseline MAP. Abbreviations: AUC, area under the curve; MBP, mean arterial pressure.

MBP Performance Metrics with 95% CI:

AUROC: 0.648 (95% CI: 0.614, 0.685)

Accuracy: 0.596 (95% CI: 0.573, 0.621)

Precision: 0.252 (95% CI: 0.223, 0.284)

Recall: 0.613 (95% CI: 0.558, 0.668)

F1-score: 0.357 (95% CI: 0.321, 0.393)


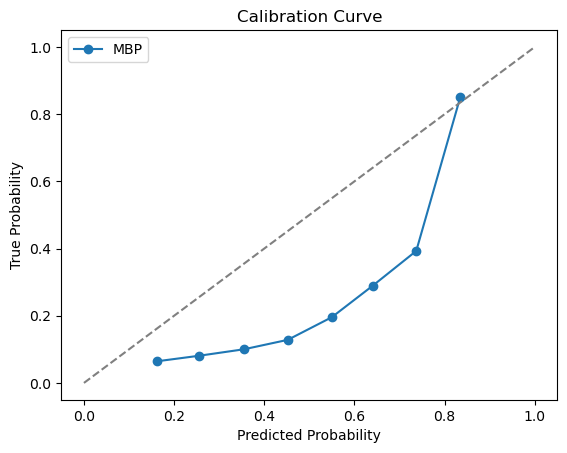


Fig. 2. Calibration curves for for the baseline MAP.

MBP Brier Score: 0.2309


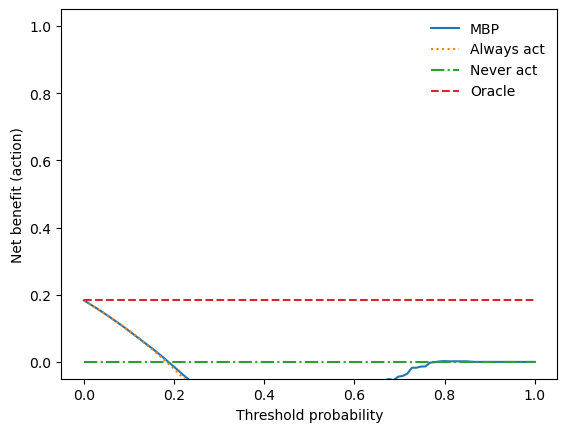


Fig. 3. Decision curve analysis for the baseline MAP.
